# Comparison of two codon optimization strategies to enhance recombinant protein production in *Escherichia coli*

**DOI:** 10.1186/1475-2859-10-15

**Published:** 2011-03-03

**Authors:** Hugo G Menzella

**Affiliations:** 1 Genetic Engineering & Fermentation Technology. CONICET. Facultad de Ciencias Bioquimicas y Farmacéuticas. Universidad Nacional de Rosario. Suipacha 531 Rosario 2000. Republica Argentina

## Abstract

**Background:**

Variations in codon usage between species are one of the major causes affecting recombinant protein expression levels, with a significant impact on the economy of industrial enzyme production processes. The use of codon-optimized genes may overcome this problem. However, designing a gene for optimal expression requires choosing from a vast number of possible DNA sequences and different codon optimization methods have been used in the past decade. Here, a comparative study of the two most common methods is presented using calf prochymosin as a model.

**Results:**

Seven sequences encoding calf prochymosin have been designed, two using the "one amino acid-one codon" method and five using a "codon randomization" strategy. When expressed in *Escherichia coli*, the variants optimized by the codon randomization approach produced significantly more proteins than the native sequence including one gene that produced an increase of 70% in the amount of prochymosin accumulated. On the other hand, no significant improvement in protein expression was observed for the variants designed with the one amino acid-one codon method. The use of codon-optimized sequences did not affect the quality of the recovered inclusion bodies.

**Conclusions:**

The results obtained in this study indicate that the codon randomization method is a superior strategy for codon optimization. A significant improvement in protein expression was obtained for the largely established process of chymosin production, showing the power of this strategy to reduce production costs of industrial enzymes in microbial hosts.

## Background

Industrial enzymes, included those used in food industry, are now traded as commodity products and there is a continuing need to reduce manufacturing costs in order to remain competitive in the global markets. *Escherichia coli *is a preferred host for the production of recombinant proteins because it combines fast growth rate, inexpensive fermentation media and well understood genetics; and the cost of production in this microorganism depends in large part upon the protein expression levels [1,2].

Species-specific variations in codon usage are often cited as one of the major causes impacting protein expression levels [3,4]. The presence of rare codons, which are correlated with low levels of their cognate tRNAs species in the cell, can reduce the translation rate and induce translation errors with a significant impact on the economy of the production process [5,6]. In the past decade, a high number of genes have been re-designed to increase their expression level [1-3,7-10]. However, designing a gene for optimal expression requires choosing from a large space containing a vast number of possible DNA sequences. Typically, two strategies have been used for codon optimization. The first one, known as "one amino acid-one codon", assigns the most abundant codon of the host or a set of selected genes to all instances of a given amino acid in the target sequence [4,8,11-15]. The second one, designed here "codon randomization", uses translation tables, based on the frequency distribution of the codons in an entire genome or a subset of highly expressed genes, to attach weights to each codon. In this case, codons are assigned randomly with a probability given by the weights [2,7,8,16,17].

Many transgenic proteins expressed in *E. coli *are recovered as insoluble aggregates in the form of inclusion bodies. The formation of these aggregates seems to be independent of the type of protein, and this drawback has been proven difficult to overcome [18,19]. Nevertheless, the fact that inclusion bodies are easy to isolate and mainly composed by the over-expressed protein facilitates product recovery at industrial scale [20,21]. Thus, the production of recombinant proteins as inclusion bodies represent a cost-effective alternative for enzyme manufacturing, provided that efficient large scale refolding methods are available. This is the case for calf prochymosin, the precursor of chymosin, which is widely used in cheese making and mostly obtained from recombinant microorganisms [18,22].

It has been recently shown that proteins contained in inclusion bodies possess a degree of secondary structure and exhibit biological activity in many cases [23-28]. Moreover, the quality of the inclusion bodies, determined by the degree of folding of the aggregated proteins, depends on different factors; many of them related to the translation rate of the corresponding mRNA [23,24]. In addition, it has been recently demonstrated that synonymous codon replacement is not always silent [29]. Codon optimization affects translation rate which, in turn, may alter protein structure and function and thus the efficiency of refolding of proteins recovered from inclusion bodies [29-31]. Therefore, codon-optimized genes may lead to the formation of inclusion bodies from which the recovered proteins could be difficult to refold.

In this study, calf prochymosin was used as a model to perform a quantitative and qualitative evaluation of the inclusion bodies obtained from the expression of a set of synthetic genes encoding this protein. Seven genes were designed and synthesized using two different codon optimization strategies, and the amount of recombinant protein and the refolding yield of the inclusion bodies obtained with each gene were compared.

## Results

### Gene design, synthesis and expression vector construction

Analysis of the native calf prochymosin gene revealed that almost 59% of codons are not the preferred for *E. coli *(Table 1). For example, seven out of the eight codons encoding for arginine found in the native sequence are represented with a frequency below 6% in the *E. coli *genome. Six of these codons are AGA or AGG, which have been shown to cause low levels of expression and mistranslational errors [1,32]. Thus, it was surmised that codon optimization of the calf prochymosin gene might result in an increase in protein expression.

**Table 1:** Codon distribution of V0, V1, V2 and wild type (WT) sequences.

AA	Codon	f^a^	WT	V0	V1	V2	AA	Codon	f^a^	WT	V0	V1	V2
Ala	GCG	0.36	1	17	17	6	Leu	CUG	0.50	23	29	29	16
	GCC	0.27	15	0	0	5		UUA	0.13	0	0	0	0
	GCA	0.21	0	0	0	4		UUG	0.13	0	0	0	5
	GCU	0.16	1	0	0	2		CUC	0.10	5	0	0	4
								CUU	0.10	1	0	0	4
Arg	CGC	0.40	1	8	0	4		CUA	0.04	0	0	0	0
	CGU	0.38	0	0	8	3							
	CGG	0.10	0	0	0	1	Lys	AAA	0.76	6	15	15	9
	CGA	0.06	1	0	0	0		AAG	0.24	9	0	0	6
	AGA	0.04	1	0	0	0							
	AGG	0.02	5	0	0	0	Met	ATG	1.00	9	9	9	9
													
Asn	AAC	0.55	11	15	15	8	Phe	UUU	0.57	6	19	0	11
	AAU	0.45	4	0	0	7		UUC	0.43	13	0	19	8
													
Asp	GAU	0.63	3	23	0	14	Pro	CCG	0.53	3	16	16	9
	GAC	0.37	20	0	23	9		CCA	0.19	1	0	0	2
								CCU	0.16	1	0	0	3
Cys	UGC	0.56	3	6	6	3		CCC	0.12	11	0	0	2
	UGU	0.44	3	0	0	3							
							Ser	AGC	0.28	13	35	0	9
Gln	CAG	0.65	24	25	25	17		UCG	0.15	4	0	0	5
	CAA	0.35	1	0	0	8		AGU	0.15	4	0	0	5
								UCC	0.15	9	0	0	5
Glu	GAA	0.69	2	14	14	10		UCU	0.15	4	0	35	6
	GAG	0.31	12	0	0	4		UCA	0.12	1	0	0	5
													
Gly	GGC	0.41	15	31	0	11	Thr	ACC	0.44	13	24	24	10
	GGU	0.34	2	0	31	10		ACG	0.27	2	0	0	6
	GGG	0.15	13	0	0	5		ACU	0.16	4	0	0	4
	GGA	0.11	1	0	0	5		ACA	0.13	5	0	0	4
													
His	CAU	0.57	4	6	6	3	Trp	UGG	1.00	4	4	4	4
	CAC	0.43	2	0	0	3							
							Tyr	UAU	0.57	5	22	0	13
Ile	AUU	0.51	2	22	0	13		UAC	0.43	17	0	22	9
	AUC	0.42	19	0	22	8							
	AUA	0.07	1	0	0	1	Val	GUG	0.37	14	26	0	10
								GUU	0.26	3	0	26	7
								GUC	0.22	7	0	0	4
								GUA	0.15	2	0	0	5

^a ^Relative frequency of each codon in *E. coli *W3110

Sequences V0 and V1 were designed using the one amino acid- one codon method; and V2 was designed with the codon randomization algorithm.

Seven variants of the calf prochymosin gene were designed using two different codon optimization strategies. In the first approach, only one codon was assigned for each amino acid to create two sequences named V0 and V1. For the V0 gene, the preferred codon found in the entire genome of *E. coli *W3110 was assigned to each amino acid. For the design of the V1 gene, a similar strategy was used; but in this case the favorite codon found in a set of highly expressed genes was employed to encode for each amino acid.

The second strategy used for codon optimization consisted on randomly assigning a triplet for each amino acid using a preference table http://www.kazusa.or.jp/codon/cgi-bin/showcodon.cgi?species=316407, with a probability based on the weight of each codon within the set encoding a given amino acid. Using this algorithm, five sequences were independently designed using the GeMS software package [16], and named V2-V6. The codon distribution for sequences V0, V1 and V2 is shown in Table 1 and the sequences of the seven genes and their codon usage is shown in Additional file 1.

The codon-optimized synthetic genes were created by using single strand 5´phosphorylated complementary primers. In all the cases 27 primers with a length ranging from 38 to 42 bases were used to create the leading strand and 27 primers with a length between from 38 to 43 bases were used to create the lagging strand. For all the genes the designed single stranded oligonucleotides overlapped each other by a minimum of 18 bases to ensure annealing. Two TAA stop codons in tandem were added at the end of each coding region followed by an *Eco*RI site. Additionally, an *Nde*I site overlapping the initial ATG was included in all the genes to mobilize the ORFs. Finally, all the synthetic genes were inserted into the expression vector pBru [17], where gene expression is driven by the P_BAD _promoter, inducible by the addition of L-arabinose.

### Protein expression and yield

**Figure 1:**
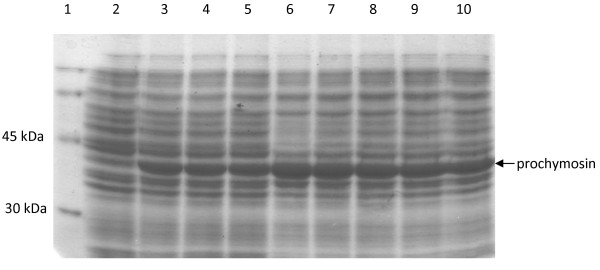
**Expression analysis of the synthetic gene variants by SDS-PAGE**. Lane 1, molecular weight marker. Lane 2, lysate of *E. coli *W3100 culture harboring the pWT expression vector for the expression of wild type calf prochymosin gene grown in the absence of L-arabinose; lanes 3-10, lysate of *E. coli *W3100 culture harboring the pV0, pV1, pV2, pV3, pV4, pV5 and pV6 expression vector for the expression of V0-V6 synthetic versions of calf prochymosin grown in the with 2 g/l of L-arabinose. In all cases, cell cultures were brought to OD_600 _= 3 and 20 μl were used for the analysis.

Recombinant plasmids were transformed into the *E. coli *W3110 strain for expression tests. Cell cultures were induced by the addition of L-arabinose when OD_600 _reached 0.5 and cells were harvested after 5 h. As previously reported, analysis of soluble and insoluble fractions of cell lysates by SDS-PAGE showed that all detectable prochymosin was located in the insoluble fraction in the form of inclusion bodies [18,22]. The amount of prochymosin produced by the expression of each gene variant was quantified by densitometry of the stained gels and is shown in Figure 1 and Table 2. The variants V2-V6 optimized by the codon randomization approach produced significantly more proteins than the native sequence. The best result was obtained for the V2 sequence with an increase of 70% in the amount of prochymosin accumulated. On the other hand, no significant improvement in protein expression was observed for the V0 and V1 variants. In both cases the amount of prochymosin produced was similar to that obtained upon the expression of the wild type gene. Inclusion bodies were washed and isolated and the yield of recombinant protein was also measured by the Lowry method [33]. Consistent with the results obtained from gel scanning quantification, the yields for V2 and the wild type sequence were 490 mg/L and 282 mg/L respectively. As reported by other authors, no correlation was found between the codon bias, measured using the codon adaptation index [34], and the quantity of recombinant protein produced by the codon-optimized genes [2,35].

**Table 2:** Amount of prochymosin produced by *E coli *W3110 cells expressing gene variants created using different codon optimization methods.

Sequence - expression vector	Codon optimization method	CAI^a^	Prochymosin produced (mg/l)^b^	Prochymosin produced (relative to wild type)
Wild type - pWT	-	0.66	262 ±19	1
V0 - pV0	One amino acid- one codon	1	248 ± 17	0.95
V1 - pV1	One amino acid- one codon	0.82	246 ± 21	0.94
V2 - pV2	Codon randomization	0.72	448 ± 31	1.71
V3 - pV3	Codon randomization	0.70	366 ± 26	1.50
V4 - pV4	Codon randomization	0.72	330 ± 16	1.36
V5 - pV5	Codon randomization	0.73	311 ± 20	1.29
V6 - pV6	Codon randomization	0.74	309 ± 29	1.21
V2/0 - pV2/0	Hybrid construct	0.97	296 ± 12	1.13

**^a ^**Codon adaptation index.

**^b ^**Determined by scanning and densitometry analysis of stained gels. Values shown represent mean and standard error of three independent experiments.

Cells were grown at 30°C in supplemented LB medium to OD_600 _= 0.5 and induced by the addition of 2 g/l of L-arabinose for 5 h.

Cell growth rates were determined for *E. coli *cells harboring each expression plasmid. No significant difference was observed among the growth curves of the recombinant strains indicating that none of the genes had a toxic effect. As expected, the growth rate of the strain harboring the empty vector was higher after the addition of the inducer (data not shown).

It has been reported that the 5'coding region is particularly important in modulating translation initiation [2,35-37]. Predicted mRNA secondary structures did not correlate strongly with expression when the sequences were analyzed using the online RNA folding program "mfold" http://mfold.rna.albany.edu/[38]. In order to investigate whether the reason for the low expression of the V0 variant was due to the presence of a 5' local element, the first 10 codons of this gene were replaced by those of the V2 gene; the variant showing the greatest level of prochymosin production. Expression of the chimeric gene V2/0 showed a modest increase in prochymosin production (Table 2), indicating that downstream elements account for the lower production of prochymosin observed for the V0 variant.

In order to investigate whether a further increase in protein expression level could be obtained by using a stronger promoter, an additional experiment was carried out. For this, the V2 sequence was cloned into the pET 24b expression vector, where the expression is driven by the T7 promoter. The resulting plasmid was transformed into the BL21(DE3) strain and three colonies grown on LB medium. In all the cases a fall in the optical density with a concomitant increase in the viscosity of the cultures was observed within 6 h after the inoculation, indicating cell lysis. This observation suggests a toxic effect of the V2 gene when controlled by the strong T7 promoter.

### In vitro refolding efficiency

Codon optimization affects translation rate which, in turn, may alter protein structure and function. It has been described that inclusion bodies formed in *E. coli *under different expression conditions may differ in their quality and, therefore, their ability to yield active proteins [25,39,40]. To the best of my knowledge, the impact of codon optimization on the quality of inclusion bodies has not been previously studied. Thus, I decided to investigate the ability of inclusion bodies obtained from the expression of different gene variants to yield functional prochymosin.

Inclusion bodies were prepared from cultures of *E. coli *W3110 strain harboring expression plasmids for the seven prochymosin gene variants described above. In all cases, inclusion bodies were washed after cell lysis and recovered as a white paste. The paste was dissolved in 8 M urea and the total protein concentration adjusted to 20 g/L. For all the preparations, PAGE analysis showed that more than 90% of the protein contained in the urea solution corresponds to calf prochymosin (data not shown). The urea solution was rapidly diluted into refolding buffer supplemented with 0.5 M arginine and 10 μM Cu^++ ^since these additives were previously shown to increase the refolding efficiency of prochymosin [18].

**Figure 2:**
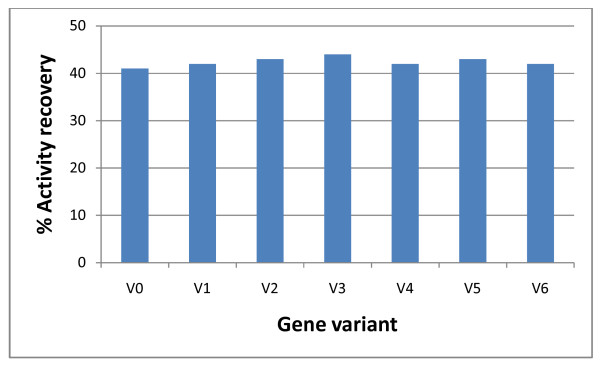
**Refolding efficiency of prochymosin prepared by expressing codon-optimized genes in *E. coli *W3110**. Renaturation was carried out by diluting the urea-solubilized inclusion bodies in renaturation buffer at a final protein concentration of 1 mg/ml and incubating the mixture at 4°C for 12 h. Other experimental details are provided in the methods section. Values shown are means of three independent determinations. The standard deviations were in all the cases less than 10% of the corresponding means.

Figure 2 shows the refolding efficiency obtained for the different inclusion body preparations. No significant differences were found in the recovery of calf prochymosin for inclusion bodies prepared using the different gene variants. In the refolding method employed, air oxidation was used to promote the formation of disulphide bonds and the activity recovered was similar to that previously reported for the native sequence [18,41]

## Discussion

Codon optimization of the calf prochymosin gene was chosen due to the commercial value of improving its expression and to study the impact of codon optimization in an established production process. Even when the wild type sequence has been reported to express well in *E. coli *[18,22], the presence of some rare codons led me to investigate codon optimization strategies in order to increase the expression of this protein. In the present study, seven gene variants were designed and synthesized to evaluate the effect of the two most common gene design strategies on the production of calf prochymosin in *E. coli*. The five sequences designed using the codon randomization strategy yielded higher protein quantities than those designed with the one amino acid-one codon method. These results suggest that the former method is a superior strategy for codon optimization. In addition, codon randomization permits flexibility in codon selection to facilitate gene design by avoiding: (i) repetitive elements that may lead to gene deletions, (ii) internal Shine-Dalgarno sequences, (iii) secondary mRNA structures and (iv) unwanted restriction sites. Some of these advantages of the codon randomization over the one amino acid-one codon method have been previously highlighted by Villalobos and coworkers [4]. However, no studies have been conducted comparing these methods side by side and many authors still propose synthetic gene-based production improvements using the one amino acid-one codon method [4,8,11-15].

Sequences V2-V6 were designed based on a codon usage table obtained from the entire genome of *E. coli *W3110. The analysis of the codon distribution of these sequences shows that the differences in expression among these genes cannot be explained by the random assignation of rare codons when using the codon randomization method (see Additional file 2). Recently, Welch and co-workers have shown that most favorable codons are those read by tRNAs that are most highly charged during amino acid starvation rather than those that are most abundant in the genome [2]. Using a codon table created based on these findings may provide genes to further increase calf prochymosin expression in *E. coli*.

No differences in protein production were found between V0 and V1 sequences, where the one amino acid-one codon algorithm using different codon tables was used for the design. This result suggests that, when employing this method, the translation efficiency may be limited by other constraints rather than the choice of the favorite codon to encode a given amino acid in the designed gene. The deleterious effect on gene expression of an imbalanced tRNA pool, previously proposed by several authors, is a likely explanation [4,42,43]. All the experiments described in this study were conducted using the P_BAD _promoter to drive the expression of the synthetic genes. Attempts to increase the level of recombinant protein using the stronger T7 promoter resulted in early lysis of the cells. A likely explanation for this observation is that the higher translation rate of the redesigned genes, associated with the leaky repression of the *lac *based T7 system in the abscense of inducer, may result in early accumulation of recombinant protein which prevents the healthy growth of the cultures. The finding of significant levels of prochymosin production in the absence of L-arabinose for *E. coli *harboring the V2 sequence supports this hypothesis.

Synonymous codon replacement may influence protein structure and function indicating that protein folding is DNA sequence dependent [24,29,44]. Polypeptides entrapped in *E. coli *inclusion bodies exhibit a variable degree of folding organization under different production conditions [19,24]. Such degree of folding is frequently correlated with the "quality" of inclusion bodies because it has an impact on the yield of refolding and, therefore, the overall economy of the production process [45,46]. This led me to explore the impact of using codon-optimized sequences on the ability of the resulting inclusion bodies to yield active chymosin. The refolding efficiency obtained for inclusion bodies recovered from recombinant clones expressing the seven individual variants were very similar, suggesting that the tested DNA sequences did not alter the conformational quality of protein contained in the inclusion bodies.

Variations in the rate of mRNA translation may influence the formation of secondary structures in the nascent polypeptide [47,48], and analysis of gene sequences and the structure of their encoded proteins show that frequently used codons are associated with structural elements, while strings of less used codons tend to be present in boundaries separating such elements. Thus, a redesigned gene where most abundant codons are placed to encode secondary structures (like alpha helices) and rare codons are placed to encode linkers, may lead to the formation of inclusion bodies of superior quality. In the redesigned genes tested in this work, codon assignment frequency was equally distributed all along the entire gene. A calf prochymosin gene designed taking into account the sequence/structure relationship may provide insights into the influence of codon optimization on the refolding efficiency. This work is currently in progress in our laboratory.

## Conclusions

Two alternative strategies for codon optimization have been evaluated in *E. coli *using calf prochymosin as a model. In all the cases the sequences created using the codon randomization method provided significantly more protein than their counterparts designed with one amino acid-one codon strategy, suggesting that this is a superior method for codon optimization. One of the obtained sequences produced more than 70% prochymosin than the native sequence, showing the potential of the approach to considerably reduce the production cost of well established production processes like in the case of chymosin.

## Methods

### General

Enzymes were obtained from New England Biolabs (USA) and used as recommended. DH5α, BL21(DE3) and W3110 *E. coli *strains were made chemically competent with a kit from Zymo Research (USA). Oligonucleotides were from Operon (USA). NTPs were PCR-grade from Roche Applied Sciences. DNA sequencing was performed on an ABI 3730 DNA analyzer (Applied Biosystems, USA) according to the manufacturer's recommended protocol. All other reagents were obtained from Sigma (USA)

### Codon optimization, gene synthesis and cloning

Seven versions of the calf prochymosin A gene were designed and constructed. The variant V0 was designed using the software GeMS [16] and a codon table containing only the most abundant codon found in the entire genome of *E. coli *W3110 for each amino acid. The variant V1 was designed using the one amino acid-one codon algorithm from the Optimizer software [42]. In this case, the favorite codon found in a set of highly expressed genes was used to encode each amino acid. Variants V2-V6 were designed using a codon randomization algorithm with the GeMS software and a codon table containing a fractional preference for each codon equal to that found in the genome of *E. coli *W3110. DNA sequences were synthesized using the method described by Reisinger and co-workers [49], digested with *Nde*I and *Eco*RI, inserted into the expression vector pBru [17] or pET 24b and verified by sequencing. In all the cases, *E. coli *DH5α was used for cloning.

### Culture growth, calf prochymosin expression

*E. coli *W3110 cells harboring the expression vectors were grown with agitation in 1 l erlenmeyer flaks containing 100 ml of Luria-Bertani medium supplemented with glycerol (10 g/l) kanamycin (50 mg/l) at 30°C. Protein expression was induced when OD_600 _reached 0.5 units by adding 2 g/l L-arabinose and incubation continued for an additional 5 h period. Final OD_600 _was typically between 8-10 units. Cells were then harvested by centrifugation at 10,000g for 15 min at 4°C.

### Protein analysis and in vitro refolding of calf prochymosin

Harvested cells (1 g wet weight) were resuspended in 40 ml of Tris-HCl 50 mM (pH 8.0) and incubated at 37^°^C for 30 minutes in the presence of lysozyme (0.2 mg/ml final concentration). Then, the mixture was sonicated on ice for 5 min. with 5 s pulses. Total extracts and proteins in different fractions were separated by SDS-PAGE on 10% gels, stained with Sypro-red and quantified by densitometry using a Typhoon scanner and BSA as a standard.

The inclusion bodies were isolated from the lysates by centrifugation at 10,000 g for 20 min at 20°C, washed twice with 50 ml of 10 mM EDTA (pH 8.0), 0.5% (v/v) Triton X-100 and once with 20 mM KH_2_PO_4 _(pH 7.5). Washed inclusion bodies were dissolved in deionized 8 M urea in 50 mM KH_2_PO_4 _(pH 10.5), rendering a final protein concentration of 20 mg/ml. The resulting solution was incubated with agitation for 2 h at 30°C, and centrifuged at 10,000 g for 10 min at 20°C. The preparation contained more than 95% prochymosin, as judged by SDS-PAGE. Refolding was carried out by rapid dilution of 1 ml of unfolded protein solution (20 mg/ml) in 20 ml of 50 mM KH_2_PO_4_, 0.5 M arginine, 10 μM CuSO_4 _(pH 10.5). The refolding solution was incubated for 12 h at 4°C. The renatured prochymosin was acidified to pH 2.0 with 2 M HCl and incubated 15 min at 20°C. Finally, samples were brought to pH 6.3 by the addition of 1 N NaOH and chymosin acivity measured using a milk clotting assay as previously described using authentic calf prochymosin (Sigma) as standard [18].

## Competing interests

The author is the inventor of a patent application that includes part of the work described in this paper.

## Supplementary Material

Additional file 1 **Sequences of gene variants: the full sequence of the synthetic genes described in this work is provided**. Click here for file

Additional file 2 **Codon usage table: The codon usage for genes V3-V6 is provided**. Click here for file
