# Supplementary material for: Comparison of two codon optimization strategies to enhance recombinant protein production in Escherichia coli
Source: Microb Cell Fact. 2011 Mar 3;10:15. doi: 10.1186/1475-2859-10-15 (PMC3056764; doi:10.1186/1475-2859-10-15)
Supplement: Additional file 1 — Sequences of gene variants: the full sequence of the synthetic genes described in this work is provided. [file 1475-2859-10-15-S1.DOC]

# Synthetic sequences encoding for calf prochymosin

# V0

ATGGCGGAAATTACCCGCATTCCGCTGTATAAAGGCAAAAGCCTGCGCAAAGCGCTGAAAGAACATGGCCTGCTGGAAGATTTTCTGCAGAAACAGCAGTATGGCATTAGCAGCAAATATAGCGGCTTTGGCGAAGTGGCGAGCGTGCCGCTGACCAACTATCTGGATAGCCAGTATTTTGGCAAAATTTATCTGGGCACCCCGCCGCAGGAATTTACCGTGCTGTTTGATACCGGCAGCAGCGATTTTTGGGTGCCGAGCATTTATTGCAAAAGCAACGCGTGCAAAAACCATCAGCGCTTTGATCCGCGCAAAAGCAGCACCTTTCAGAACCTGGGCAAACCGCTGAGCATTCATTATGGCACCGGCAGCATGCAGGGCATTCTGGGCTATGATACCGTGACCGTGAGCAACATTGTGGATATTCAGCAGACCGTGGGCCTGAGCACCCAGGAACCGGGCGATGTGTTTACCTATGCGGAATTTGATGGCATTCTGGGCATGGCGTATCCGAGCCTGGCGAGCGAATATAGCATTCCGGTGTTTGATAACATGATGAACCGCCATCTGGTGGCGCAGGATCTGTTTAGCGTGTATATGGATCGCAACGGCCAGGAAAGCATGCTGACCCTGGGCGCGATTGATCCGAGCTATTATACCGGCAGCCTGCATTGGGTGCCGGTGACCGTGCAGCAGTATTGGCAGTTTACCGTGGATAGCGTGACCATTAGCGATGTGGTGGTGGCGTGCGAAGGCGGCTGCCAGGCGATTCTGGATACCGGCACCAGCAAACTGGTGGGCCCGAGCAGCGATATTCTGAACATTCAGCAGGCGATTGGCGCGACCCAGAACCAGTATGGCGAATTTGATATTGATTGCGATAACCTGAGCTATATGCCGACCGTGGTGTTTGAAATTAACGGCAAAATGTATCCGCTGACCCCGAGCGCGTATACCAGCCAGGATCAGGGCTTTTGCACCAGCGGCTTTCAGAGCGAAAACCATAGCCAGAAATGGATTCTGGGCGATGTGTTTATTCGCGAATATTATAGCGTGTTTGATCGCGCGAACAACCTGGTGGGCCTGGCGAAAGCGATT

# V1

ATGGCGGAAATCACCCGTATCCCGCTGTACAAAGGTAAATCTCTGCGTAAAGCGCTGAAAGAACACGGTCTGCTGGAAGACTTCCTGCAGAAACAGCAGTACGGTATCTCTTCTAAATACTCTGGTTTCGGTGAAGTTGCGTCTGTTCCGCTGACCAACTACCTGGACTCTCAGTACTTCGGTAAAATCTACCTGGGTACCCCGCCGCAGGAATTCACCGTTCTGTTCGACACCGGTTCTTCTGACTTCTGGGTTCCGTCTATCTACTGCAAATCTAACGCGTGCAAAAACCACCAGCGTTTCGACCCGCGTAAATCTTCTACCTTCCAGAACCTGGGTAAACCGCTGTCTATCCACTACGGTACCGGTTCTATGCAGGGTATCCTGGGTTACGACACCGTTACCGTTTCTAACATCGTTGACATCCAGCAGACCGTTGGTCTGTCTACCCAGGAACCGGGTGACGTTTTCACCTACGCGGAATTCGACGGTATCCTGGGTATGGCGTACCCGTCTCTGGCGTCTGAATACTCTATCCCGGTTTTCGACAACATGATGAACCGTCACCTGGTTGCGCAGGACCTGTTCTCTGTTTACATGGACCGTAACGGTCAGGAATCTATGCTGACCCTGGGTGCGATCGACCCGTCTTACTACACCGGTTCTCTGCACTGGGTTCCGGTTACCGTTCAGCAGTACTGGCAGTTCACCGTTGACTCTGTTACCATCTCTGACGTTGTTGTTGCGTGCGAAGGTGGTTGCCAGGCGATCCTGGACACCGGTACCTCTAAACTGGTTGGTCCGTCTTCTGACATCCTGAACATCCAGCAGGCGATCGGTGCGACCCAGAACCAGTACGGTGAATTCGACATCGACTGCGACAACCTGTCTTACATGCCGACCGTTGTTTTCGAAATCAACGGTAAAATGTACCCGCTGACCCCGTCTGCGTACACCTCTCAGGACCAGGGTTTCTGCACCTCTGGTTTCCAGTCTGAAAACCACTCTCAGAAATGGATCCTGGGTGACGTTTTCATCCGTGAATACTACTCTGTTTTCGACCGTGCGAACAACCTGGTTGGTCTGGCGAAAGCGATCTGA

# V2

ATGGCAGAAATCACTCGCATCCCTTTGTACAAAGGAAAATCTCTGCGCAAGGCGCTTAAAGAACATGGGCTCCTGGAGGATTTCCTTCAGAAACAGCAATATGGTATTAGCTCTAAATACAGTGGTTTTGGCGAGGTTGCCAGCGTTCCGCTCACGAATTATCTGGACTCGCAGTACTTCGGCAAGATTTATCTGGGGACGCCGCCCCAGGAATTTACAGTACTGTTCGATACCGGAAGTTCCGACTTCTGGGTGCCATCTATTTATTGCAAGAGCAACGCCTGTAAGAATCATCAACGTTTTGACCCGCGCAAAAGTTCAACATTCCAGAACCTTGGTAAGCCCCTGTCGATTCATTACGGAACCGGCTCCATGCAAGGCATTCTGGGTTATGACACAGTGACTGTGTCCAATATTGTTGATATTCAACAAACTGTGGGCCTGAGCACCCAGGAACCAGGTGACGTATTCACGTACGCAGAATTTGATGGTATTCTGGGCATGGCGTATCCGTCTCTGGCGAGCGAATATAGCATACCGGTGTTTGATAACATGATGAACCGTCACCTGGTGGCTCAGGATCTCTTTTCGGTTTACATGGATCGCAACGGCCAGGAATCAATGCTGACGTTGGGGGCGATCGACCCGTCATATTACACGGGGAGTTTGCACTGGGTCCCGGTTACGGTGCAGCAGTACTGGCAGTTTACCGTAGATTCTGTTACCATCAGTGACGTTGTGGTGGCTTGCGAAGGCGGCTGCCAGGCAATCCTGGATACCGGCACCAGCAAACTTGTGGGGCCTAGCTCAGATATTCTCAACATTCAGCAAGCCATCGGCGCCACCCAGAATCAGTATGGTGAGTTTGATATCGATTGTGATAACCTGTCGTACATGCCTACCGTCGTATTCGAAATTAATGGAAAGATGTATCCGTTGACCCCGTCAGCATATACAAGCCAGGACCAAGGTTTTTGTACTTCGGGTTTTCAGTCCGAGAATCACTCTCAAAAATGGATTCTGGGAGACGTATTCATTCGTGAATATTATTCCGTCTTTGATCGGGCGAACAATTTGGTCGGTCTGGCCAAAGCGATCTGA

# V3

ATGGCGGAAATCACTCGTATCCCATTGTACAAAGGCAAATCTCTGCGCAAAGCATTAAAAGAGCATGGGCTCCTGGAGGACTTCCTACAGAAACAGCAATACGGCATTTCCTCAAAATATAGCGGCTTCGGTGAGGTGGCATCTGTACCGTTGACCAACTATCTGGATAGCCAGTACTTTGGTAAAATTTATTTGGGCACGCCACCTCAGGAGTTCACCGTGTTGTTTGACACGGGTTCCTCAGATTTCTGGGTCCCGTCAATTTATTGTAAAAGCAACGCGTGTAAGAACCATCAGCGCTTCGATCCGCGTAAGAGTTCCACCTTTCAAAACCTGGGCAAACCGCTGTCCATTCATTACGGCACAGGCTCAATGCAGGGCATTTTGGGCTACGACACGGTTACTGTAAGCAATATTGTTGATATCCAGCAAACCGTGGGACTGTCCACGCAAGAGCCGGGAGATGTTTTCACATATGCGGAATTCGACGGTATCCTGGGAATGGCCTACCCGAGCCTGGCGAGCGAATACTCGATCCCGGTCTTTGACAACATGATGAATCGCCATTTAGTCGCGCAGGATTTATTTTCCGTGTATATGGACCGAAATGGCCAGGAGAGTATGCTGACCTTAGGGGCAATCGACCCAAGTTACTACACCGGTTCATTACACTGGGTTCCAGTGACGGTCCAACAGTATTGGCAATTCACCGTGGATAGCGTCACTATCAGCGACGTGGTTGTAGCATGCGAGGGCGGTTGCCAGGCGATTCTGGACACTGGGACGAGCAAACTCGTGGGGCCGAGCTCTGACATTTTGAACATCCAACAAGCGATCGGAGCGACCCAGAATCAATACGGGGAATTTGATATTGATTGCGATAACCTGTCTTATATGCCGACCGTCGTTTTCGAGATAAACGGGAAGATGTACCCGCTGACTCCGTCCGCGTATACATCTCAAGACCAGGGCTTTTGCACCTCCGGCTTTCAATCTGAAAATCACTCTCAAAAATGGATATTGGGGGATGTTTTCATTCGTGAGTACTACAGCGTGTTCGATCGTGCTAATAACCTTGTGGGACTCGCGAAAGCAATC

# V4

ATGGCAGAGATAACCCGTATTCCTCTCTACAAAGGCAAATCCCTGCGAAAAGCGCTGAAAGAGCATGGCTTGTTAGAGGACTTTCTGCAGAAACAGCAGTATGGGATTAGTTCTAAATACTCTGGTTTTGGCGAAGTTGCCTCTGTTCCGCTAACGAATTACTTAGATAGCCAGTATTTCGGTAAAATCTACCTGGGCACTCCCCCGCAAGAATTCACCGTATTATTCGACACCGGGAGCTCCGATTTCTGGGTCCCGAGCATTTACTGTAAAAGCAATGCGTGCAAGAACCACCAACGT
TTTGATCCTCGCAAGTCCTCAACGTTTCAGAACCTGGGGAAACCTCTTTCAATACACTATGGTACGGGAAGTATGCAGGGCATTCTCGGATACGATACTGTGACGGTTAGCAATATCGTCGACATTCAACAGACGGTTGGGCTTTCCACACAAGAACCGGGTGATGTCTTCACGTACGCCGAATTTGACGGCATTCTGGGCATGGCTTATCCGAGCCTGGCATCGGAGTACTCGATTCCTGTTTTTGATAACATGATGAATCGTCATCTGGTAGCGCAGGATCTGTTTAGCGTTTACATGGACCGTAACGGCCAGGAATCAATGTTAACCCTGGGTGCGATTGATCCAAGTTATTACACGGGTTCCCTGCATTGGGTCCCTGTAACCGTGCAGCAATACTGGCAGTTCACTGTTGATTCC
GTAACCATTTCAGATGTAGTGGTTGCGTGCGAAGGGGGCTGCCAGGCCATTCTGGACACGGGCACCTCTAAACTGGTGGGGCCGAGCAGCGATATTCTGAACATCCAACAAGCAATTGGGGCGACCCAGAACCAGTATGGGGAGTTCGACATCGATTGCGACAACCTGTCGTATATGCCAACCGTGGTGTTCGAAATCAATGGCAAAATGTACCCTCTGACCCCGTCGGCGTACACCAGCCAAGATCAAGGTTTCTGCACGTCGGGTTTCCAGAGCGAAAACCACTCTCAAAAGTGGATTTTGGGCGATGTTTTCATCCGCGAATATTATTCGGTGTTTGATAGAGCCAATAACCTGGTGGGCCTCGCAAAAGCTATT

# V5

ATGGCGGAAATAACTCGCATTCCTCTCTATAAAGGTAAATCGCTCCGCAAAGCTTTAAAGGAACACGGCCTTCTGGAGGATTTTCTGCAGAAGCAGCAGTACGGCATCAGTAGCAAATATTCTGGTTTTGGTGAGGTAGCGTCCGTACCGTTAACCAACTACCTGGATAGTCAGTATTTTGGCAAGATCTACCTGGGTACCCCGCCTCAGGAATTTACCGTGCTCTTTGATACCGGCAGCTCTGATTTCTGGGTTCCGTCAATCTATTGTAAATCGAATGCTTGTAAAAATCATCAGCGCTTTGATCCGCGCAAATCTAGCACCTTTCAAAACCTCGGCAAGCCCCTGTCGATCCATTACGGTACCGGTTCAATGCAGGGCATCCTGGGCTATGATACGGTAACGGTGAGTAACATTGTGGACATTCAACAAACTGTAGGGCTGTCCACTCAAGAACCAGGTGATGTGTTCACCTATGCAGAATTTGACGGCATTCTGGGCATGGCGTACCCGTCGCTGGCGAGCGAGTATAGTATTCCGGTGTTCGACAATATGATGAACCGCCACTTGGTTGCCCAGGATCTGTTCTCCGTCTATATGGATCGTAATGGGCAGGAGAGCATGCTGACCTTGGGTGCGATTGATCCGAGTTATTATACCGGTAGCCTGCACTGGGTACCGGTTACGGTTCAGCAGTATTGGCAGTTCACCGTGGATAGCGTTACCATAAGCGACGTTGTGGTCGCCTGTGAGGGCGGTTGTCAGGCCATACTGGATACGGGGACCAGCAAACTGGTGGGGCCGTCGTCAGATATTCTGAATATTCAGCAGGCCATTGGAGCGACGCAAAACCAATACGGTGAATTCGATATTGATTGCGATAACCTCAGCTATATGCCCACGGTAGTGTTCGAGATTAACGGCAAAATGTATCCCCTTACCCCCTCGGCTTATACAAGTCAAGACCAAGGGTTCTGCACCTCGGGCTTTCAGTCAGAGAATCATAGCCAGAAGTGGATTCTTGGCGATGTATTCATCCGTGAGTATTACAGTGTTTTTGACCGTGCTAACAACCTGGTCGGGCTTGCGAAGGCGATT

# V6

ATGGCAGAAATCACGCGGATTCCTCTGTATAAAGGCAAATCGCTGCGTAAAGCCTTAAAAGAACATGGCCTGCTGGAGGATTTCCTGCAAAAGCAGCAGTATGGCATCAGCAGTAAATACTCTGGCTTTGGCGAAGTTGCGTCGGTCCCGCTGACCAACTATTTGGACTCTCAGTACTTTGGCAAAATCTATCTCGGTACACCACCGCAAGAATTCACGGTTCTCTTTGATACCGGCAGCTCCGATTTTTGGGTGCCGTCTATCTATTGCAAGTCGAATGCATGCAAAAACCATCAGCGCTTTGACCCACGCAAAAGCAGTACATTTCAGAACCTCGGGAAGCCGCTGTCGATCCATTACGGCACCGGTAGTATGCAGGGTATCTTGGGTTATGACACTGTGACCGTCTCGAACATAGTGGATATCCAACAAACCGTGGGATTAAGTACTCAGGAACCAGGGGACGTTTTCACTTACGCGGAATTCGATGGTATCCTGGGCATGGCATACCCGAGCTTGGCTAGCGAATACAGCATCCCGGTGTTTGACAATATGATGAATAGACATTTAGTGGCGCAAGATTTATTCTCTGTGTACATGGATCGCAACGGCCAGGAATCAATGTTGACTCTGGGTGCCATCGATCCGTCCTACTATACGGGATCACTGCACTGGGTGCCAGTGACCGTTCAGCAGTACTGGCAGTTTACCGTGGACTCGGTGACAATCTCAGATGTGGTGGTGGCATGCGAAGGCGGATGTCAAGCAATCCTGGATACCGGCACCTCCAAACTCGTGGGCCCGAGTTCCGATATCCTGAACATCCAACAGGCGATCGGTGCCACCCAGAATCAGTACGGCGAATTTGACATTGACTGTGATAATCTGTCATACATGCCGACGGTAGTATTCGAAATTAATGGAAAAATGTACCCGCTTACCCCGAGTGCTTATACGTCACAAGATCAAGGCTTCTGTACCTCCGGTTTCCAAAGCGAAAACCACAGTCAAAAATGGATTCTGGGCGACGTGTTCATCCGCGAGTACTATTCAGTGTTCGATCGCGCAAACAACCTGGTGGGCCTGGCGAAGGCAATT
